# Conformal Integration of Efficient Conductive-Ink-Printed Antennas in Smart Suitcases for LPWAN-Based Luggage Tracking

**DOI:** 10.3390/s22114077

**Published:** 2022-05-27

**Authors:** Igor Lima de Paula, Hendrik Rogier, Patrick Van Torre

**Affiliations:** Internet Technology and Data Science Lab (IDLab), Department of Information Technology, Ghent University-Imec, 9052 Ghent, Belgium; hendrik.rogier@ugent.be (H.R.); patrick.vantorre@ugent.be (P.V.T.)

**Keywords:** antenna design, conformal sensors, coplanar waveguide, flexible substrates, Internet of Things, low-power wide area network (LPWAN), printed electronics, smart devices

## Abstract

In the context of localization and sensing within the Internet of Things, new antenna manufacturing technologies, such as antennas printed with conductive inks on thin thermoplastic sheets, allow for seamless integration into plastic objects produced by an injection molding process. In this paper, we present printed sensor antennas for the [862–928] MHz band supporting LoRa and Sigfox and the [2.4–2.5] GHz band for WiFi, Bluetooth, and IEEE802.15.4 communication. To integrate them into smart suitcases, the antennas are printed, overmolded, tested, and measured, following a dedicated conformal integration strategy consisting of two design iterations. Additionally, as a more convenient connection to the printed antennas, printed transmission lines along with a dedicated transition to printed circuit board technologies are implemented and characterized, avoiding rigid coaxial connectors that exhibit fragile mounting on flexible substrates. The overmolded stand-alone antennas achieve fractional impedance bandwidths of 26% and 15% covering the [862–928] MHz and [2.4–2.5] GHz bands, respectively, with a substantial margin and with in-band simulated total efficiencies of 94% and 88%, respectively. Finally, the seamless integration of two antennas into a smart suitcase for tracing via Sigfox and WiFi demonstrates the potential of the proposed technique to realize high-performance antennas occupying virtually no real estate.

## 1. Introduction

The ever more ubiquitous Internet of Things (IoT) is set to support an unprecedented number of interconnected devices with services [1] that permit new communication, localization, and sensing applications and revolutionize many aspects of our daily life [2]. The key enablers are the emerging low-power wide area networks (LPWANs) provided by technologies such as LoRa and Sigfox. They allow devices, typically requiring a low data rate, to establish long-range links at low power levels [1,2,3] while benefiting from a wide coverage area [2] and enhanced reliability. Therefore, LPWAN technologies will potentially replace dedicated networks in industry automation, agriculture, smart grids, and logistics tracking [4], lowering capital expenditures and the operation costs of communication infrastructure.

A market that could benefit from IoT services is the airline transportation industry [5]. Despite the increased efforts expended on luggage tracking, airlines worldwide saw a rise of 2.2% in luggage mishandling in 2018, as a consequence of the growing capacity pressure experienced before the COVID-19 pandemic; moreover, the passenger volume is expected to double by 2037 [6]. Luggage is currently tracked by a multitude of technologies, such as optical systems, barcodes, radio frequency identification (RFID), or manual interventions [6], complicating the flow of information between handlers. Although RFID has been proposed for luggage tracking in several works [7,8,9] and recommended by the International Air Transport Association (IATA) [10], it requires investments in private infrastructure by the operators and is based on checkpoints throughout the luggage trajectory. Other possible solutions have also been considered, including Bluetooth [11], a combination of global positioning system (GPS) and short message service (SMS) technologies [12,13], and impulse radio ultra wideband (IR-UWB) technology [14]. Nevertheless, suitcase tracking could be improved in terms of coverage, reliability, and interoperability by relying on emerging low-power wide area network technologies.

Therefore, we propose a scheme employing a combination of the Sigfox Monarch localization service [3,15] and WiFi sniffing [5,16,17]. In this paradigm, once landed, a smart suitcase takes advantage of the available WiFi networks to triangulate its position within tens of meters. The Sigfox Monarch feature is able to identify the country or territory [3,15] where the suitcase is located, serving as a fall-back solution for WiFi sniffing. Then, the suitcase reports its location several times a day using the widely available Sigfox network, which is independent of individual airport implementations, in contrast to RFID-based systems. In this way, the luggage is tracked using technology available across operators worldwide. This facilitates the employment of non-disposable antennas, which are more robust because their integration platform can be accounted for in the design stage. This work outlines, applies, and validates a new integration technique involving the design of antennas printed on thin polymer sheets [18] and overmolded into plastic objects during the injection molding process. The main contribution of this paper is a novel design-for-integration cycle, where antennas are designed, simulated, implemented, measured, overmolded, and remeasured. The shift in properties due to overmolding is compensated for by means of a redesign, such that the second similar design cycle results in a properly working overmolded antenna. The antenna is realized in a single metal layer that is seamlessly integrated into the plastic surface of a suitcase hardshell. The planar platform allows for large antennas to be realized while occupying virtually no real estate, leading to highly efficient, wideband designs.

Previously, the authors of [7,14] overlaid an RFID tag and an IR-UWB antenna on a suitcase shell, resulting in increased robustness and reusability compared to the currently used disposable RFID tags. More generally, antenna integration was demonstrated with a handbag by reusing its metallic zipper as a radiator [19]. Other designs were implemented on the body of a smartwatch [20], on tiles for smart floors [21], and on smart surfaces made of virtually any material [22], such as medium-density fiberboard (MDF).

Since the IoT terminal is usually deeply integrated into these smart devices, which are made of diverse materials [23], the antenna system designer must judiciously consider the influence of this integration on its reliable performance and the goal of increased battery life [24]. Major hurdles are the frequency detuning and communication link impairment caused either by the electromagnetically unpredictable nature of the smart device materials or by objects surrounding the smart device. In [22], an alternative is proposed by building substrate-independent antennas that rely on an air-filled cavity-backed antenna directly fabricated on the smart device. Although highly efficient substrate-independent microwave components were created with this concept [22], the smart device shell is not always thick enough to implement it; in addition, only one metal layer [25,26] may be available. In [23], an active reconfigurable antenna system is selected that electronically shifts the resonance frequency of a narrowband antenna to accommodate for detuning. Although the high Q-factor antenna makes miniaturization possible [23], it comes at the cost of increased complexity and extra power loss for the added active circuits. Finally, the work in [21] copes with detuning by adopting a large design margin on the impedance bandwidth.

Antennas have been integrated into objects before, but this integration often did not unify the antennas with these objects to the same degree that is possible with current large-scale manufacturing technologies. Here, by taking the influence of the suitcase material into account during the design stage, two antenna topologies are demonstrated with a −10-dB impedance bandwidth covering the Sigfox [862–928] MHz band and the WiFi ISM [2.4–2.5] GHz band with a large margin, having percentile bandwidths of 26% and 15%, respectively, and achieving in-band simulated total efficiencies of at least 94% and 88%, respectively.

The conformal integration strategy is further outlined in Section 2. Specific details related to the design of the smart suitcase building blocks are described in Section 3. Next, the measured results of the fabricated prototypes for the stand-alone antennas and the antennas overmolded in a suitcase hardshell are presented in Section 4, together with a pertinent discussion. Finally, Section 5 concludes this paper.

## 2. Conformal Integration Strategy

To create a technological platform enabling the seamless and invisible integration of antennas and transmission lines into smart objects, we apply a dedicated additive manufacturing process that prints passive radio frequency (RF) devices employing conductive ink on a thin thermoplastic foil, which is then completely integrated into the polymer shell of an object. We demonstrate this with a suitcase shell, in which the antennas are overmolded during the same process used to cast standard suitcase shells by injecting molten plastic at high pressure into a suitcase shell mold [27]. Consequently, absolute integration is obtained, such that the RF devices become an indivisible part of the shell, also conforming to its shape and texture. This allows for relatively large antennas to be realized without compromising real estate. To reduce manufacturing costs and complexity, only one metal layer is employed, in which transmission lines are realized using coplanar waveguide (CPW) technology. This also ensures the compatibility of our design and manufacturing procedure with thin plastic shells.

**Figure 1:**
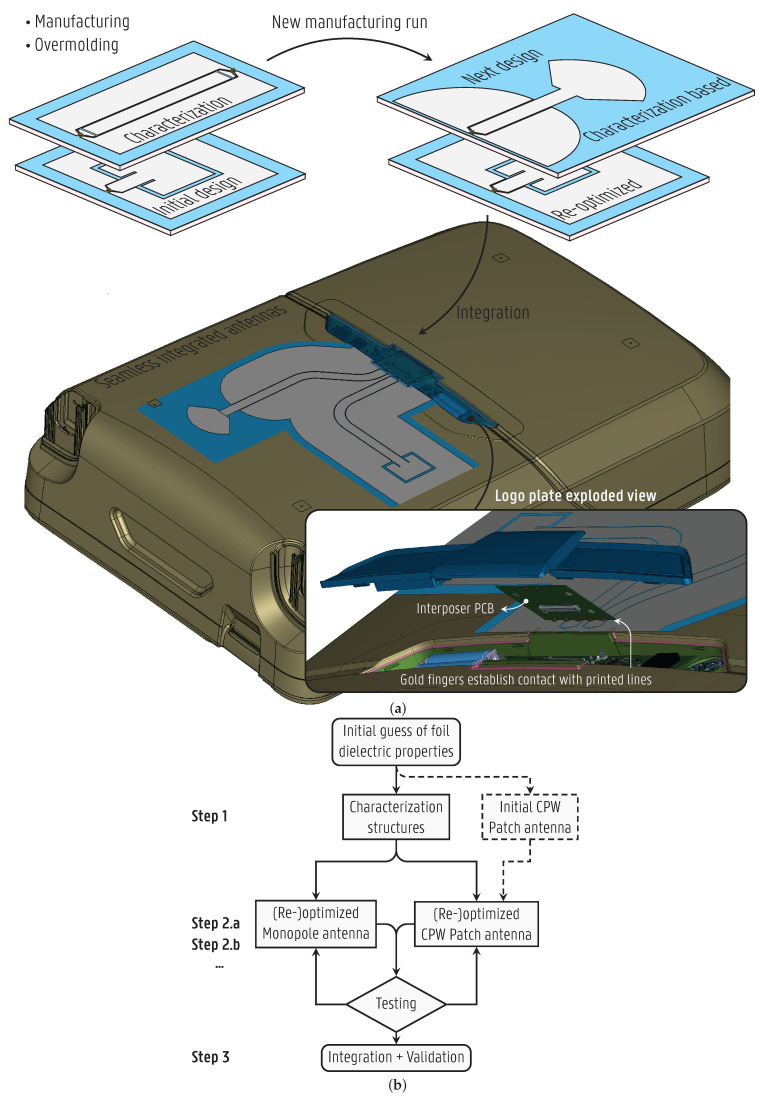
Design-for-integration process a as key enabler for a coplanar waveguide (CPW) patch and a CPW monopole antenna, covering the [2.4–2.5] GHz WiFi band and [862–928] MHz Sigfox band, respectively, seamlessly integrated into a suitcase shell. (**a**) Overview. The antennas are printed on a thermoplastic foil that is overmolded into the suitcase shell at high pressure at its casting resulting in seamless integration (see Figure 2). Inset: exploded view of suitcase’s logo plate, housing the radio frequency (RF) modules and the dedicated interposer printed circuit board (PCB), which interconnects the printed antennas. (**b**) Flowchart.

The design flow to achieve high-performance integrated antennas is outlined in the flowchart in Figure 1b. In the first step, the test structures are designed based on an initial guess of the dielectric properties, i.e., $\epsilon_{r}$ and tan δ, of the polypropylene (PP) foil, whereas the effects of the overmold PP material are ignored at this stage. The test structures are then manufactured and overmolded in a PP slab to characterize the dielectric properties of both the foil alone and the resulting overmolded substrate that is consistent with the final application. Step 2 involves designing and fine-tuning the antennas thoroughly in a full-wave simulation environment based on the knowledge acquired from the material characterization. The optimized antennas are tested after fabrication and overmolding. In case their resonance frequencies lie out of the operating band, the additional information provided by the measurements serves to re-optimize the antenna dimensions in a subsequent iteration of step 2. As an alternative path, an initial antenna is designed and fabricated together with the test structures, also based on the initial guess of the foil’s dielectric properties and neglecting the overmolding effects. Following this approach, the initial antenna’s measured results serve to confirm the characterization or to directly re-optimize the antenna, compensating for the frequency shift introduced by the substrate materials. After successfully realizing the stand-alone overmolded prototypes, the antennas are integrated in the smart object in step 3. At this point, both antennas are positioned within the available surface on the suitcase, and their feeds are extended, connecting to the RF modules. Such removable and replaceable modules are housed in the suitcase logo plate, where a dedicated interposer printed circuit board (PCB) establishes contact with the printed CPW lines through gold fingers, as illustrated in the inset of Figure 1a. In the final application, the highly efficient antennas are driven by ultra-low-power active circuits [28,29] to maximize battery life. By reducing the data rate to the minimum, we enable the system to be powered without batteries through energy harvesters (e.g., indoor solar cells) and supercapacitors.

## 3. Design Aspects

### 3.1. Platform Materials and Characterization

**Figure 2:**
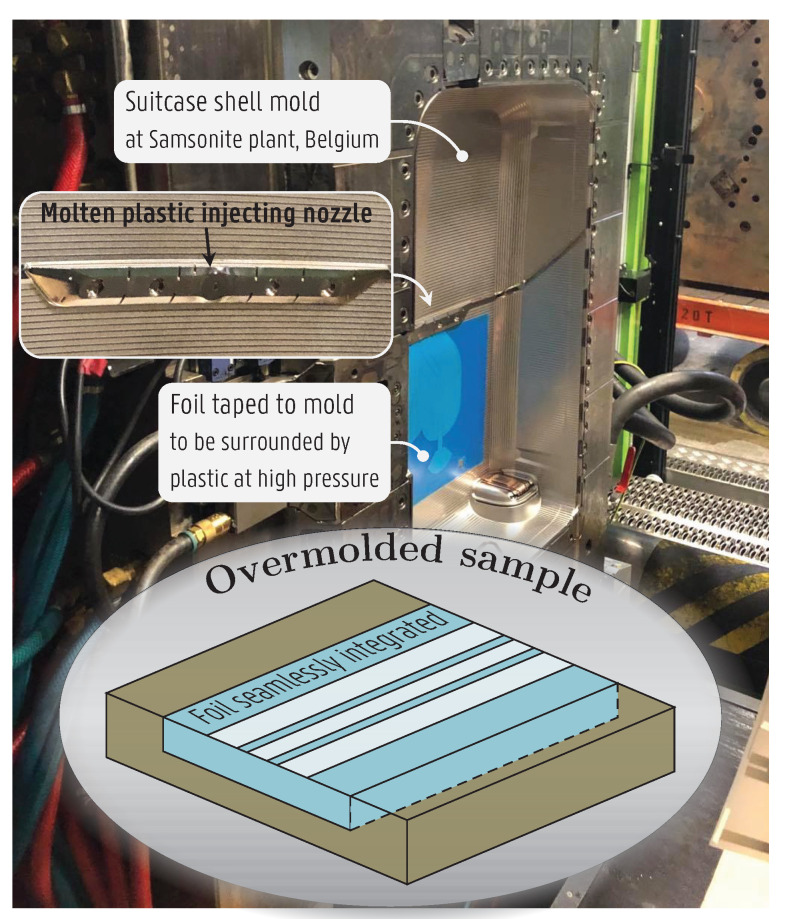
Illustration of the overmolding process. The foil carrying the antenna is taped to the suitcase shell mold, where molten plastic is injected at high pressure resulting in the foil being seamlessly integrated into the plastic shell.

The adopted conformal integration platform consists of a 150-μm-thick PP foil, serving as a carrier on which the antennas and microwave devices are printed using a 4-μm-thick silver-based ink with conductivity $\sigma =6.8\cdot 10^{6}\;S/m$ supplied by Quad Industries [30]. The foil is coated with polyurethane (PU) for improved ink adhesion. The carrier is overmolded on a suitcase whose shell is made of 2.7-mm-thick PP. The overmolding, resulting in a seamless integration of the foil into plastic objects, is achieved by sticking the foil to a mold into which molten plastic is injected at high pressure and high temperature, as depicted in Figure 2. The foil is surrounded by the plastic, except at the printed face (see inset of Figure 2), becoming one with the plastic object. After integration, no air gaps are present between the printed antennas and the suitcase shell; they form a single monolithic smart object. The materials and the procedure are specifically designed in order for the antenna to stay intact during this fabrication process.

**Figure 3:**
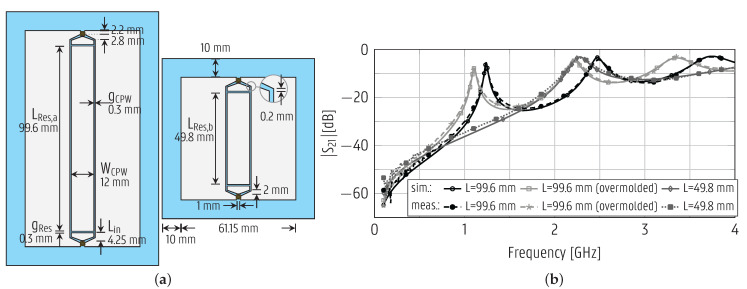
Substrate characterization resonators. (**a**) To-scale representation. The defined parameters are the same for both resonators, unless otherwise specified. (**b**) Magnitude of the transmission coefficient.

A specific design strategy is needed to develop antenna systems with optimal radiation characteristics after full integration into the suitcase shell. Therefore, in the first step of this process, the electromagnetic properties of the foil and the overmolded suitcase shell material were characterized by designing and fabricating two line resonators [31] of different lengths, as indicated in Figure 3a. After manufacturing, the resonators were assembled with U.FL connectors [32] using conductive glue. An instance of the longer resonator line (left of Figure 3a) was overmolded in the suitcase shell material. The prototypes were measured by an Agilent E8364B vector network analyzer (VNA), and the measurement cables were de-embedded. The measurements were fitted onto full-wave simulations by Keysight’s Advanced Design System (ADS) Momentum 3D Planar EM Simulator to obtain the $\epsilon_{r}$ and the tan δ. The measured and simulated curves achieve excellent agreement, as shown in Figure 3b, when the PP foil has as dielectric properties [$\epsilon_{r}\;$=6.03, tan δ= 0.02] and the overmolded material has as material properties [$\epsilon_{r}\;$=1.8, tan δ= 0.019] in the [0–4] GHz frequency range.

A 108.7-mm long CPW transmission line was also fabricated and measured to yield an insertion loss of 0.24 dB at $f_{1}$ = 895 MHz and 0.93 dB at $f_{2}$ = 2.45 GHz. As for the overmolded transmission line, the measured insertion loss was 0.30 dB at $f_{1}$ = 895 MHz and 0.68 dB at $f_{2}$ = 2.45 GHz. By relying on the good agreement between the measured curves and simulation results obtained from ADS, a CPW line made of lossless media was simulated and leveraged to estimate the contribution of each loss mechanism. The lossless media in the simulation comprised the perfect electric conductor (PEC) metal layer, as well as the foil and overmolded dielectric materials with permittivities of $\epsilon_{r}\;$=6.03 and $\epsilon_{r}\;$=1.8, respectively. In Table 1, the insertion loss of the CPW lines is decomposed into components originating from reflection, radiation, and metal/dielectrics losses. This decomposition corresponds to subdividing the two-port network, being the CPW line, into three cascaded subnetworks representing each contribution, with each having its own insertion loss. The computation of these components is specified in Table 1 in terms of the S-parameters measured (meas.) for the CPW line, i.e., $S_{11}$ and $S_{21}$, and those of the simulated (sim.) model based on lossless media, i.e., $S_{11}^{ll}$ and $S_{21}^{ll}$. Note that the insertion loss of the CPW line in the absence of reflections is given by $-10\log_{10}|S_{21,\mathrm{reflectionless}}{|}^{2}=-10\log_{10}\left(\right|S_{21}{|}^{2}+|S_{11}{|}^{2})$. The larger insertion loss observed for the non-overmolded prototype at 2.45 GHz, attributed to the increased reflection losses, occurs because the CPW line does not behave as an ideal transmission line. Its characteristic impedance varies considerably according to the electrical length, such that its physical length must be optimized for the band of operation to mitigate reflections.

For comparison, a 1.37-mm low-loss U.FL coaxial patch cord exhibits an insertion loss of about 1.4 dB/m at 868 MHz and 2.2 dB/m at 2.45 GHz [32], corresponding to 0.15 dB and 0.24 dB, respectively, for a length of 108.7 mm. Despite the higher losses of the CPW line, out of several material combinations compatible with thermoforming, the selected materials exhibited the best adhesion and direct current (DC) performance before and after overmolding. Furthermore, the values indicate that the CPW line is a good alternative for short connections where the integration of a coaxial cable is difficult to achieve, as in thin thermoplastic surfaces. Note that even a U.FL coaxial cable is very thick compared to our printed CPW transmission lines.

### 3.2. Conductive-Ink-Printed Antennas

The smart suitcase that demonstrates our proposed integration technique must be equipped with antennas providing communication in the range of [862–928] MHz (hereafter referred to as the 895 MHz band), i.e., the combined short-range device (SRD) 860 MHz and industrial, scientific and medical (ISM) 915 MHz bands, allowing both LoRa and global SigFox communication. The hardware should also support the [2.4–2.5] GHz ISM band, enabling WiFi sniffing for geolocalization, Bluetooth, or IEEE802.15.4 communication. It is desirable that the antennas are matched to 50Ω with a −10-dB impedance bandwidth at least three times wider than the operating bands. The additional bandwidth is important because substantial detuning due to the items in the suitcase is expected. Overspecification by adding wide margins to the impedance bandwidth is the strategy to mitigate such detuning. Additionally, since smart devices are typically battery-powered, we aim for a total radiation efficiency greater than 90% for the overmolded stand-alone antennas in free space. The topology is constrained to a single metal layer to reduce cost and increase compatibility with device shells of various thicknesses. This uniplanar topology is also compatible with the process and material thickness variation involved in the overmolding, as detailed in Section 3.3.

**Figure 4:**
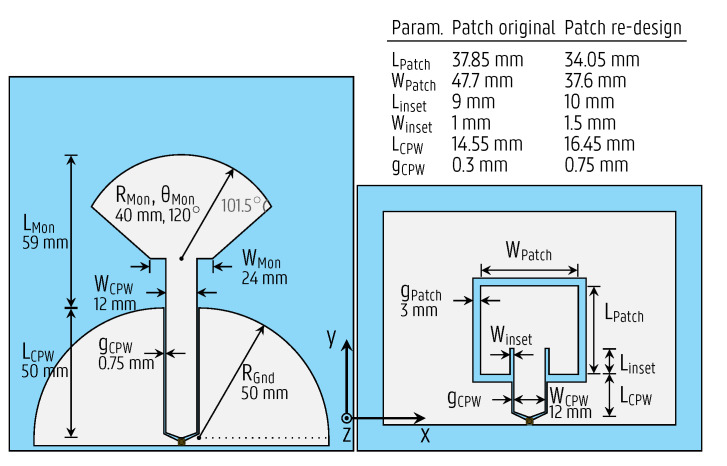
To-scale representation of the printed antennas. Left: Monopole covering the [862–928] MHz Sigfox band; right: coplanar patch covering the [2.4–2.5] GHz WiFi band. The table lists the values for the defined parameters, which were modified during the coplanar patch antenna redesign, whereas the other parameter values are indicated below their labels.

As the second step of the design process, to fulfill these requirements while ensuring the optimal characteristics for integration into smart objects, a different antenna topology is chosen for each band, as depicted to scale in Figure 4. A radial monopole [33] is chosen for the 895 MHz band, and a coplanar patch antenna [34] (also known as a slot loop [35] and folded-slot antenna [36]) for the 2.45 GHz ISM band. The monopole is initially considered to have a length $L_{\mathrm{Mon}}$, defined in Figure 4, given by $L_{\mathrm{Mon}}=\lambda_{g}/4$, where $\lambda_{g}$ is the guided wavelength [33]. However, the radial tip of the monopole capacitively loads the stub, leading to a miniaturization compared to the initial size. The radial tip also broadens the bandwidth, since the fringing fields around it dissipate energy through radiation, thus decreasing the Q-factor. For the CPW patch antenna, the slot loop circumference should be approximately equal to the guided wavelength, i.e., $2\left(W_{\mathrm{Patch}}+L_{\mathrm{Patch}}\right)=\lambda_{g}$ [36]. By limiting the length $L_{\mathrm{Patch}}$, low cross-polarization levels can be maintained, which also reduces the coupling between both antennas.

Both antennas are simulated in an electromagnetic field solver, CST Microwave Studio, and fine-tuned to achieve broadband impedances around their respective center frequencies. The miniaturized monopole has the advantage of providing a more compact antenna design relative to its wavelength; it is only 31% larger than the CPW patch antenna despite having a wavelength 2.7 times larger. Therefore, the monopole is chosen for the 895 MHz band. To showcase the design procedure and the manufacturing process with another antenna topology, the CPW patch antenna is chosen for the 2.45 GHz band; it is small enough for the application at its operating band and typically less sensitive to detuning by nearby objects. Yet, apart from the aforementioned arguments, both antennas could be implemented in an identical topology during production for simplicity and cost reasons.

### 3.3. Overmolded Antennas

The antennas are to be overmolded in their actual deployment scenario, seamlessly integrated into plastic objects as described in Section 3.1. Although the antenna stays intact during the overmolding process, the dielectric properties of the plastic are expected to influence the resonance frequencies of the antennas. By bringing this dielectric material (relative permittivity er > 1) in close vicinity of the antenna, the effective guided wavelength at the center frequency $f_{0}$ becomes smaller. Thereby, the ratio of the antenna dimensions with respect to the wavelength increases, lowering the resonance frequency. Yet, since the proposed antennas are uniplanar, consisting of a single metal layer, the field patterns at the resonance frequency are not primarily concentrated in the dielectric substrate. Instead, they are also distributed in the air surrounding the antennas, such that the effective relative permittivity, $\epsilon_{\mathrm{eff}}$, which is the weighted average permittivity experienced by the antennas, and the corresponding guided wavelength, are not heavily dependent on the antenna substrate’s dielectric properties or thickness. Acknowledging that the overmold leads to a frequency shift, the antennas are designed for correct operation in the intended frequency band after overmolding. This is achieved by designing the stand-alone antenna prototypes (Figure 4) in step 2 of the process flow (see Figure 1), considering the effects of overmolding by characterizing the substrate dielectric properties, as executed in step 1. More specifically, the characterized dielectric property constants are applied to a full wave simulation environment, such as CST Microwave Studio, to accurately model the PP foil and the overmold material, while adjusting the antenna dimensions for optimal performance at $f_{0}$. If necessary, in a subsequent iteration of step 2, a redesign is then performed to update the antenna dimensions based on the measured resonance frequency, $f_{0,\mathrm{meas}}$, of the antenna prototype by leveraging the formula $d_{\mathrm{new}}=d_{\mathrm{old}}f_{0,\mathrm{meas}}/f_{0}$, where $d_{\mathrm{old}}$ and $d_{\mathrm{new}}$ are the original and updated relevant dimensions of the antennas, respectively.

An alternative path is also demonstrated for the CPW patch by skipping step 1 of the process flow and initially fabricating the original CPW patch antenna without prior characterization of the substrate materials. Next, in an iteration of step 2, a redesign is performed to compensate for the frequency shift.

### 3.4. Interfacing the Antennas

Interfacing the RF signals originating on a PCB to printed antennas can be challenging. Either a rigid connector or a printed transmission line, with adapted feed, can be used. Both approaches have been studied and implemented.

#### 3.4.1. U.FL Coaxial Connector

The most straightforward way of interfacing with the printed antennas is by using a lightweight connector to a coaxial cable with a certain flexibility. Therefore, during the prototyping stage, we applied U.FL connectors [32], which are compact and can be attached to the printed antennas by conductive glue. This interfacing method was adopted for the measurements of the stand-alone antennas, characterization resonators, and transmission line prototypes. However, the mechanical fragility of the assembly remains an issue. Although proper connections to the prototype were realized and their performance validated, unplugging a cable often detaches the U.FL connector, breaking the prototype’s printed conductive ink. This demonstrates why interfacing the antennas with U.FL connectors directly bonded to them is not expected to deliver the necessary mechanical stability for the final application under conditions of vibration and repetitive shocks.

#### 3.4.2. Interposer to Printed Transmission Line

**Figure 5:**
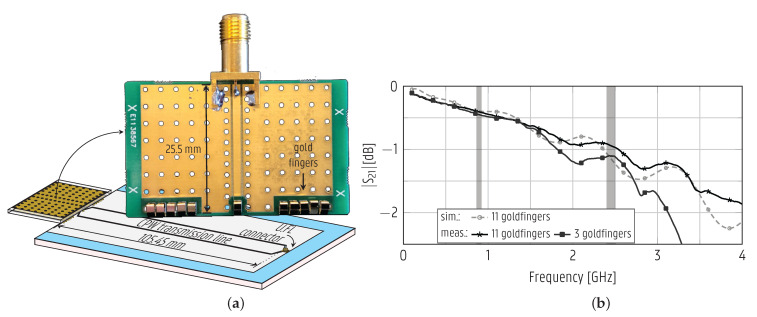
Interposer PCB used to connect to the antennas. (**a**) The measurement setup to characterize the insertion loss of the interposer PCB contacting a CPW line and (**b**) the corresponding simulated and measured magnitude of the transmission coefficient. Prototypes with 3 and 11 gold fingers were fabricated and measured.

To create a robust and reliable interface with the printed antennas and to simplify the assembly at the mass-manufacturing stage, a dedicated interposer PCB was developed. This interposer PCB is displayed in Figure 5a. As visible in the figure, the interposer PCB implements the connection to a printed transmission line via a number of gold finger contacts press-fit onto the printed transmission line. This eliminates the delicate process of gluing a connector to the printed RF devices. The interposer PCB can be fabricated through standard PCB manufacturing processes and screwed onto the logo plate of the suitcase (see Figure 1) to establish a reliable connection to the overmolded antennas. To characterize such an interfacing technique, a printed transmission line was tested in combination with an interposer PCB by bridging a subminiature version A (SMA) connector to the printed transmission line. The insertion loss of an interposer PCB interfaced with a 105.45-mm-long CPW line printed on a thermoplastic foil was measured using the setup illustrated in Figure 5a. The gold fingers touch the conductive ink of the ground and signal track. The $|S_{21}|$ curve of the interposer PCB shown in Figure 5b was measured by a VNA from the SMA connector at the interposer PCB to the U.FL connector at the printed CPW line. The measurement for the interposer with 11 gold finger contacts corresponds well to a simulation performed in CST Microwave Studio. After this measurement, the eight outermost gold finger contacts were removed, leaving only the center three contacts. Figure 5b shows that the three gold finger contacts appear sufficient for operation both in the 895 MHz and 2.45 GHz ISM bands. The use of a dual-channel interposer PCB is demonstrated in Section 4.3 to feed both the monopole and CPW patch antennas overmolded into the suitcase. In the final application, the interposer PCB itself is interconnected via U.FL cables to a main PCB housed inside the logo plate of the suitcase. The bonding of the U.FL connector to a rigid PCB in a standard industrial soldering process does not suffer from the fragility observed in the direct assembly of the U.FL to the printed antennas.

## 4. Antenna Measurement Results and Analysis

The performance of the monopole and patch antennas was validated by measurements of their reflection coefficients and far-field radiation patterns in an anechoic chamber, using an Agilent N5242A PNA-X VNA and an NSI-MI antenna positioning system. The front and back hemispheres of the radiation patterns presented in Section 4.1 and Section 4.2 were measured in separate steps, and the curves were joined afterwards. The calibration of the radiation patterns was performed by gain comparison with the standard gain horns MI-12-0.75 and MI-12-1.7. The measured curves were compared to the simulated counterparts obtained from the frequency-domain solver of CST Microwave studio.

### 4.1. 895-MHz Conductive-Ink-Printed Monopole Antenna

**Figure 6:**
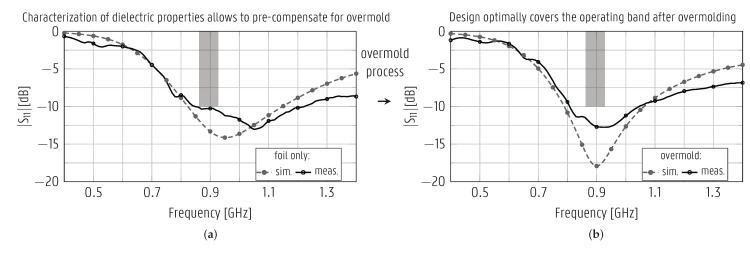
Simulated and measured magnitude of the reflection coefficient of the monopole antenna (**a**) printed on plastic foil and (**b**) after overmolding. Stand-alone prototypes fed with U.FL connector (shown on the left of Figure 4).

The monopole antenna prototypes, shown on the left of Figure 4, were optimized during step 2 of the design procedure based on the substrate characterization in step 1, as described in Section 3.1, and manufactured. They were fed through a 10-cm U.FL cable for validation. The measured and simulated magnitude of the reflection coefficient is presented in Figure 6, exhibiting a good agreement both before (Figure 6a) and after (Figure 6b) undergoing the overmolding process. Both prototypes cover the [862–928] MHz band with a large margin. Compared to the intended operating band, the measured magnitude of the reflection coefficient before (after) overmolding is below -10 dB in a 561% (371%) wider range than required, lying in the interval [850–1220] MHz ([805–1050] MHz). The robustness of the antenna against detuning by dielectrics in its proximity is confirmed since the 895-MHz band remains covered by all the prototypes.

**Figure 7:**
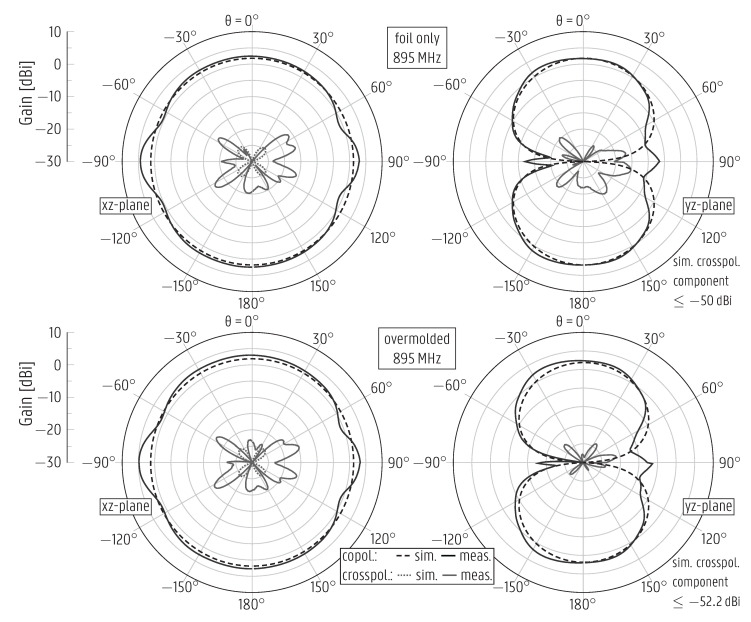
Simulated and measured radiation patterns of the monopole antenna stand-alone printed on plastic foil (**top**) and after overmolding (**bottom**).

Additionally, the measured and simulated radiation patterns, depicted in Figure 7, also agree well. Moreover, they are omnidirectional in the xz-plane, resembling the radiation pattern of an ideal dipole, since the monopole is coplanar with the ground plane. The simulated cross-polarized components remain below −24.5 dBi in the principal planes, whereas the measured values are much higher, up to −15 dBi, as seen in Figure 7. The higher values in the measurement were expected because the measured cross-polarized component is influenced by the inevitable contribution of the coaxial cable feeding the antenna.

The radiation patterns are only slightly affected by the overmolding process, with the broadside gain changing from 2.4 dBi to 3 dBi. This happens because the overmolded layer thickness is orders of magnitude smaller than the wavelength, such that most of the fields propagate in the air and, as such, the effective permittivity remains almost unaltered by adding this dielectric layer. The simulated total efficiency of the overmolded monopole is stable and above 94% in the [862–928] MHz band.

### 4.2. 2.45-GHz Conductive-Ink-Printed CPW Patch Antenna

**Figure 8:**
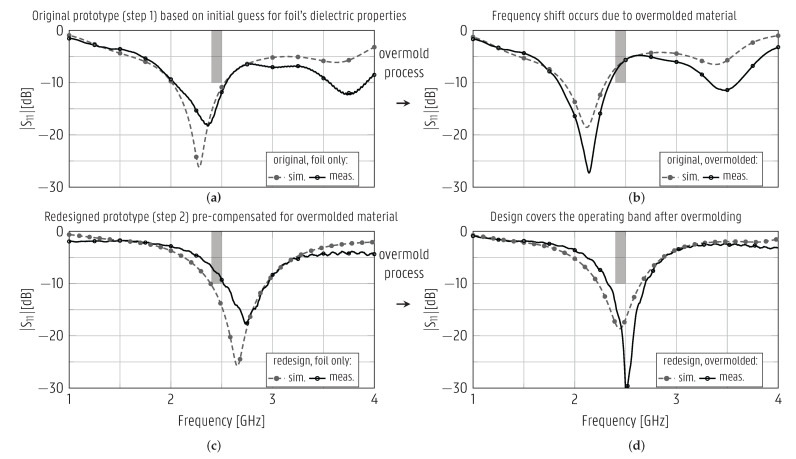
Simulated and measured magnitudes of the reflection coefficient of the original (**a**,**b**) and redesigned (**c**,**d**) patch antennas, both printed on plastic foil (**a**,**c**) and after overmolding (**b**,**d**). Stand-alone prototypes fed by U.FL connector (shown on the right of Figure 4).

The CPW patch antenna was initially designed via an alternative path in step 1 of the design procedure, before the substrate materials were characterized (see the flowchart in Figure 1b). Therefore, it is based on an educated guess of the plastic foil’s electromagnetic characteristics, $\epsilon_{r}$ = 2.9 and tan δ = 0.02, obtained from literature, whereas the effect of the overmolding material was ignored during this initial design step. Based on the information provided by this design cycle and the material characterizations presented in Section 3.1, the CPW patch antenna was re-optimized in step 2 of the design procedure, and new prototypes were manufactured. These prototypes, having the dimensions shown in the top right of Figure 4, were excited by a 10-cm U.FL cable, which was de-embedded from the measurements. Figure 8 displays the simulated and measured magnitudes of the reflection coefficient of both the original and the redesigned CPW patch antenna both before and after undergoing the overmolding process. In all cases, a good agreement between the measured and simulated curves is observed. The resonance shifted towards substantially lower frequencies after overmolding; it centered around $f_{0}$ = 2.144 GHz for the original design, and did not cover the ISM 2.45-GHz band anymore. As seen in Figure 8c,d, the redesigned antenna was optimized in step 2, such that it covered the [2.4–2.5] GHz band after overmolding. The overmolded redesigned antenna is impedance-matched to 50Ω in the band [2.32–2.69] GHz, confirming that the goal of the redesign was met.

The antenna’s large −10-dB impedance bandwidth of 370 MHz, compared to the 100 MHz bandwidth of the 2.45- GHz ISM band itself, showcases the robustness of the design to manufacturing discrepancies and to objects in close proximity of the antenna.

**Figure 9:**
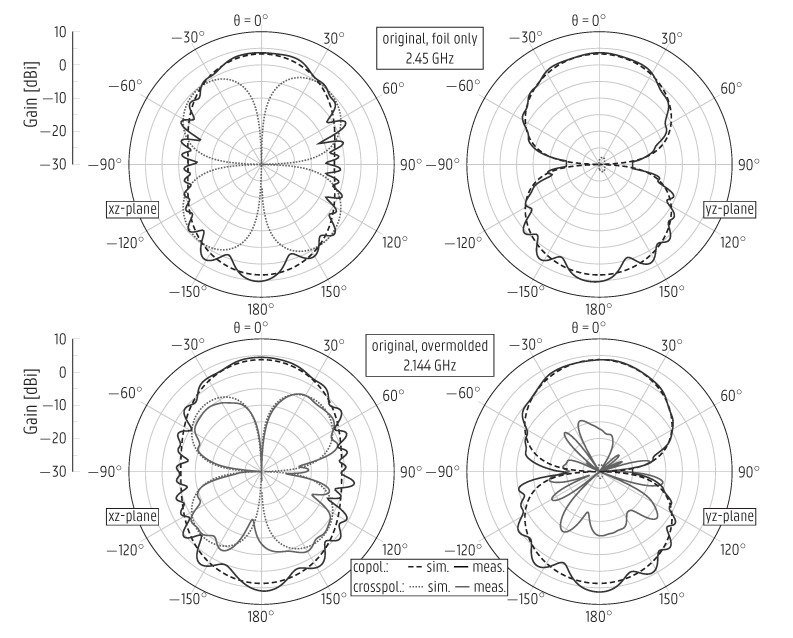
Simulated and measured radiation patterns of the original coplanar patch antenna, stand-alone printed on plastic foil (**top**) and after overmolding (**bottom**).

**Figure 10:**
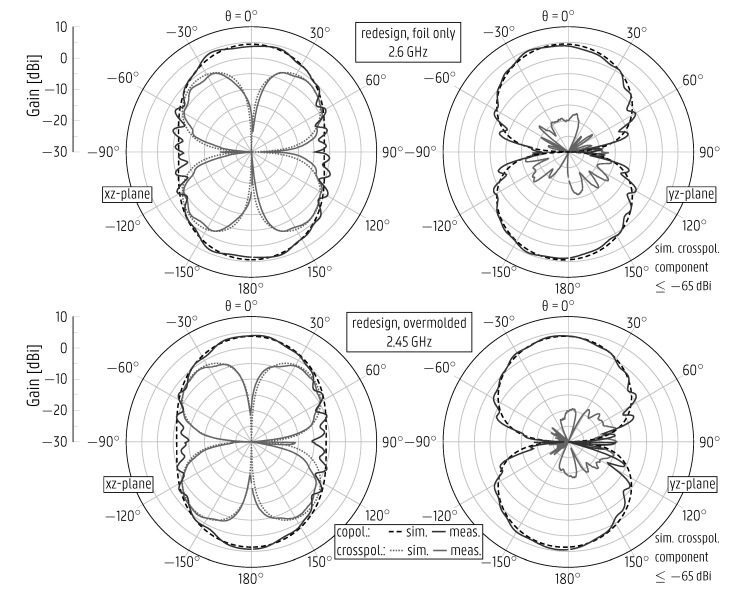
Simulated and measured radiation patterns of the redesigned coplanar patch antenna, stand-alone printed on plastic foil (**top**) and after overmolding (**bottom**).

The measured radiation patterns of all versions of the CPW patch antenna mentioned above are displayed in Figure 9 (for the original design) and in Figure 10 (for the redesign), exhibiting an excellent agreement with the respective simulated curves. The curves for the original CPW patch antenna after overmolding and for the redesigned version before overmolding are presented at 2.144 GHz and 2.6 GHz, respectively, as these prototypes are not impedance-matched at the center frequency $f_{0}\;$=2.45 GHz. The overmolded prototype’s measured gain is stable within the band of operation, [2.4–2.5] GHz, varying in the range from 4.3dBi to 4.64dBi. Due to the asymmetry around the xz-plane (Figure 4, antenna on the right), in that the bottom of the antenna differs from the top by the presence of the feedline, a strong cross-polarized component appears in the xz-plane.

By relying on the good agreement between the simulated and measured radiation patterns, the total efficiency was computed by using the directivity/gain method described in [37], based on subtracting the simulated directivity from the measured gain. The simulated total efficiency of the overmolded redesigned CPW patch antenna is at least 88% for the [2.4–2.5] GHz band, whereas the measured value amounts to 94%.

### 4.3. Monopole and CPW Patch Overmolded on Suitcase

The monopole and CPW patch antennas were redesigned with an extended feedline and overmolded into the suitcase. Since the CPW lines behave in a substantially different manner than an ideal transmission line, as described in Section 3.1, the lengths were optimized to minimize insertion loss, yielding a 147.6-mm long feedline for the monopole and a 225.5-mm long feedline for the patch. Furthermore, the CPW patch width, $W_{\mathrm{Patch}}$ (defined in Figure 4) was changed to 40mm for improved impedance matching. In the optimization process, we considered the antennas’ resonances shift towards lower frequencies when the suitcase is filled with clothes. To increase the isolation between both antennas, the CPW patch antenna was rotated by 90${}^{\circ }$ as described.

**Figure 11:**
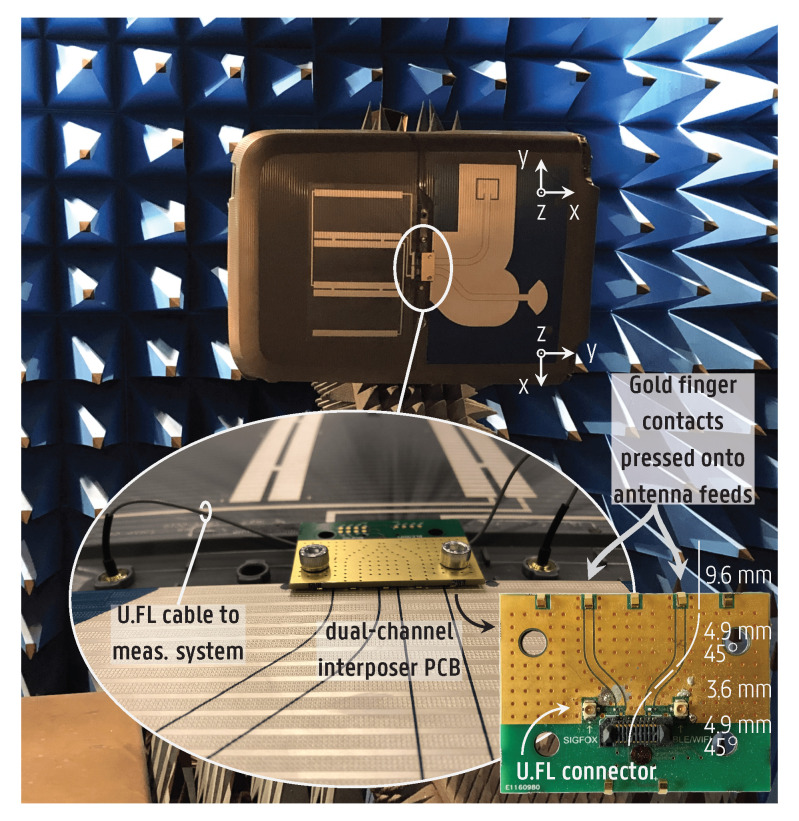
Setup used to measure the monopole and redesigned CPW patch antennas integrated by overmolding into the suitcase. The antennas are flush with the exterior of the suitcase shell. The structure on the left of the antennas is an interconnection pattern for solar cells (not implemented). Inset: dual-channel interposer PCB interconnecting the antennas. The interposer’s grounded coplanar waveguide (GCPW) lines, whose lengths are annotated, are jumpered to U.FL connectors. The coordinate system is oriented individually at each antenna, consistent with the coordinate system introduced in Figure 4.

The antennas were interconnected by a dual-channel version of the interposer PCB presented in Figure 5a, which now contains two signal tracks with a total of five gold finger contacts, as shown in the inset of Figure 11. The resulting prototype and the setup used to measure the radiation patterns of both antennas are illustrated in Figure 11, with the antennas on the right side and, on the left side, a solar panel interconnection pattern, which was not yet implemented and not connected to the antenna system. Each antenna was connected to the measurement system in turn, while the other antenna port was terminated by a 50-Ω load.

**Figure 12:**
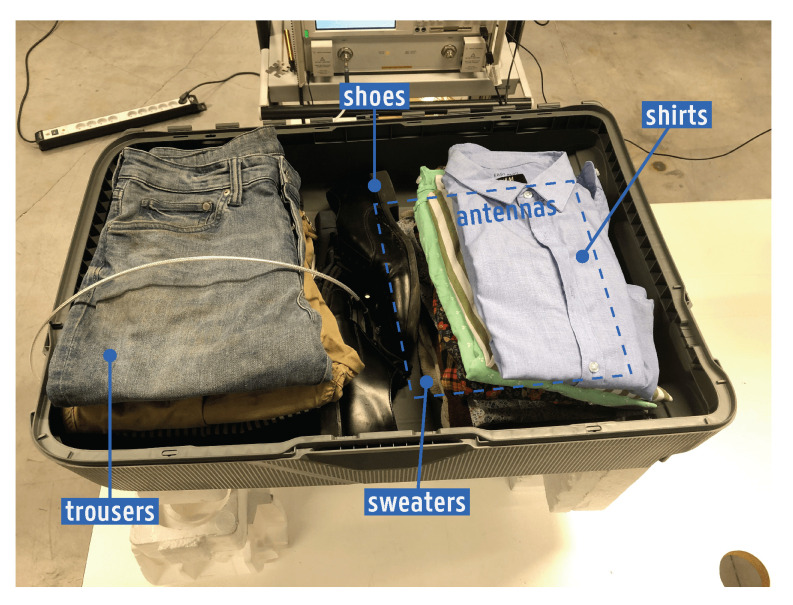
Suitcase filled with a standard set of clothes, comprised of trousers, shoes, sweaters, and shirts. This setup was used for determining the influence of the suitcase’s contents on the antennas’ reflection coefficient.

**Figure 13:**
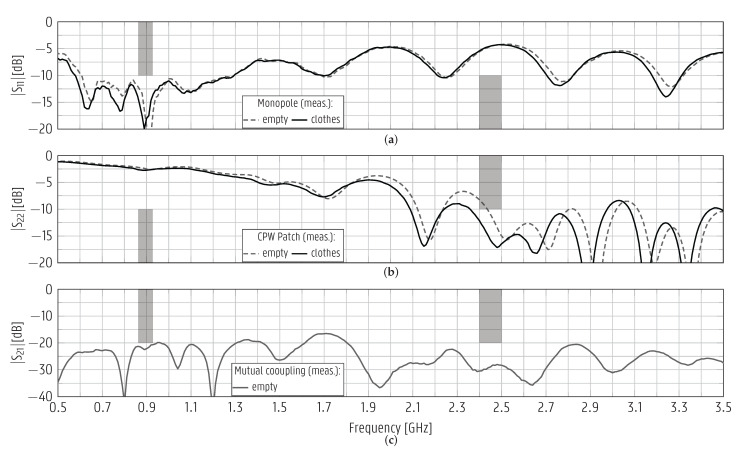
Measured (meas.) magnitudes of the reflection coefficient of (**a**) monopole and (**b**) redesigned CPW patch antennas as well as (**c**) their meas. mutual coupling. The antennas were overmolded in the suitcase and interconnected by the interposer PCB. The curves in (**a**,**b**) refer to the cases when the suitcase is empty and when it is filled with a standard set of clothes.

The reflection coefficient was measured according to the setup detailed in Figure 12, containing a standard set of clothes. The average compositions of the items right behind the antennas were 79% cotton and 21% polyester for the shirts and, for the sweaters, 55% cotton, 20% polyester, 12% wool, 11% polyamide, and 2% acrylic. The curves plotted in Figure 13 show that the monopole and the CPW patch are impedance-matched in the intended [862–928] MHz and [2.4–2.5] GHz bands. Additionally, both antennas also succeeded in covering the operating bands when the suitcase contained:

(i) only shirts;
(ii) only sweaters;
(iii) sweaters topped by a laptop.

As for an empty suitcase, which is not the targeted use case, the $|S_{11}|$ curves of the monopole and the CPW patch are below −12.0dB and −8.2dB, respectively, in the [862–928] MHz and [2.4–2.5] GHz bands. The mutual coupling between the antennas is shown in Figure 13c. Its level remains below −20 dB in the operating bands of both antennas. This significant isolation is desirable for the simultaneous operation of both antennas. It is achieved by imposing that each antenna is poorly impedance-matched in the other’s operating band. Additionally, the CPW patch antenna is rotated by 90${}^{\circ }$, such that the antennas’ strongest field components are orthogonal to each other.

**Figure 14:**
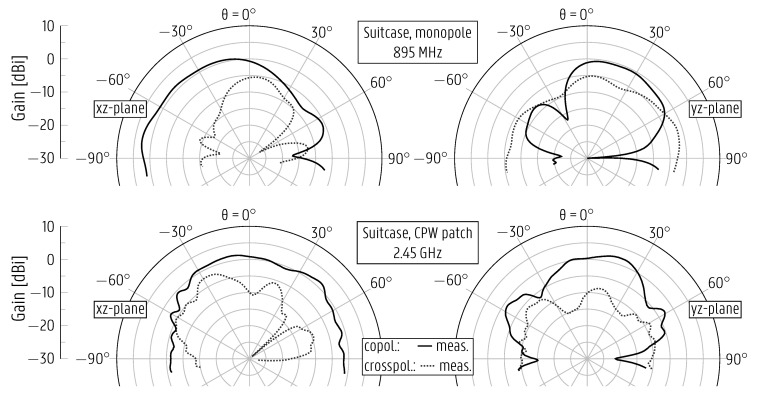
Measured radiation patterns of monopole and redesigned CPW patch antennas overmolded in the suitcase and interconnected by the interposer PCB. The coordinate system is defined individually at each antenna, as displayed in Figure 11, for consistency with the results presented in subsections Section 4.1 and Section 4.2.

Figure 14 displays the measured radiation patterns of both antennas in the xz- and yz-planes, which are defined individually for each antenna and as in Figure 11. Due to the complexity of the setup, only the frontal hemisphere was measured for the radiation pattern cuts. However, a close resemblance to the back hemisphere is expected based on the results previously presented in Figure 7, Figure 9 and Figure 10. Additionally, due to weight constraints and the difficulty in securing the clothes in place while attaching the suitcase to the rotor in the anechoic chamber, the radiation patterns were measured only for the empty-suitcase scenario. The patterns possess an asymmetry due to the mutual coupling and parasitic radiation of the other antenna when one antenna is excited, such that one antenna acts as a director for the other. This is observed in the xz-plane for the monopole and in the yz-plane for the CPW patch antenna. Furthermore, the parasitic radiation of the monopole feedline causes an asymmetry in its radiation in the yz-plane. The monopole exhibits a 3-dB beamwidth of 87${}^{\circ }$in the xz-plane frontal hemisphere, with the main beam located in the interval [3${}^{\circ }$, 90${}^{\circ }$] and a maximum gain of 2.8dBi. For the CPW patch, there is a beamwidth of 114${}^{\circ }$, with the main beam in the interval [−64${}^{\circ }$, 50${}^{\circ }$] and a maximum gain of 1.5dBi in the xz-plane.

The higher levels of the cross-polarized components as compared to those of the stand-alone antennas (Section 4.1 and Section 4.2) originate from the asymmetry introduced in the antennas after their integration. Nevertheless, the cross-polarized components are not specifically to be avoided in the current application since the polarization of the incoming waves is randomly oriented.

## 5. Conclusions

A dual-band antenna system was designed and implemented for seamless integration into the plastic shell of a suitcase, relying on a novel conformal integration strategy. Pervasive integration into the shell was achieved by overmolding a CPW monopole and a CPW patch antenna during the injection molding manufacturing process of the suitcase shells.

CPW resonators were designed by means of full-wave simulation and printed in silver ink on polypropylene foils. Additionally, a CPW patch antenna was designed and implemented based on an educated guess of the RF material properties. Resonators were employed in order to accurately determine the RF parameters of the materials. Later, the patch antenna was then overmolded in the suitcase factory. By measuring the impedance bandwidth and the radiation patterns before and after overmolding, the influence of the overmolding process was characterized. The plastic surrounding the antenna caused a downward shift of the center frequency. During the final iteration of the design process, the patch antenna was redesigned in order to compensate for the influence of the overmolding process, and an 895-MHz CPW monopole antenna was directly designed for correct operation after overmolding based on the characterization of the resonators.

For more profound integration, CPW transmission lines and a dedicated interposer PCB were designed, implemented, and characterized, providing a transition to interconnect the antennas to a removable transceiver circuit. The 895 MHz monopole and 2.45 GHz patch antennas were printed together on a single sheet with extended CPW feed lines and interfaced by the interposer PCB.

The complete suitcase with fully integrated antennas and transmission lines was measured in an anechoic chamber, fulfilling the performance requirements. The conformal integration strategy was very effective in designing and implementing an efficient antenna system for a smart suitcase, as demonstrated by the measurements. Absolute seamless integration into the suitcase shell was achieved, with, for the monopole and the patch, respectively: a −10 dB impedance matching in the [580–1300] MHz range and in the [2.36–2.98] GHz range for the suitcase filled with a standard set of clothes, and maximum gains of 2.8dBi and 1.5dBi for a frontal hemisphere beamwidth of 87${}^{\circ }$ and 104${}^{\circ }$ in the xz-plane, all measured with respect to a port plane at the interposer PCB’s connector. This antenna system is intended as a basis for a traceable suitcase using Sigfox and WiFi.

## Figures and Tables

**Table 1:** Insertion loss of CPW line decomposition.

Loss Component	Computation (*)	895 MHz	2.45 GHz
Non-overmolded CPW line			
Reflection	$-[10\log_{10}|S_{21}{|}^{2}-10\log_{10}\left(\right|S_{21}{|}^{2}+|S_{11}{|}^{2})]$	0.00dB	0.35dB
Radiation	$-10\log_{10}\left(\right|S_{21}^{ll}{|}^{2}+|S_{11}^{ll}{|}^{2})$	0.02dB	0.13dB
Metal/dielectrics	$-[10\log_{10}\left(\right|S_{21}{|}^{2}+|S_{11}{|}^{2})-10\log_{10}\left(\right|S_{21}^{ll}{|}^{2}+|S_{11}^{ll}{|}^{2})]$	0.22dB	0.45dB
Total insertion loss	$-10\log_{10}{\left|S_{21}\right|}^{2}$	0.24dB	0.93dB
Overmolded CPW line			
Reflection	$-[10\log_{10}|S_{21}{|}^{2}-10\log_{10}\left(\right|S_{21}{|}^{2}+|S_{11}{|}^{2})]$	0.04dB	0.07dB
Radiation	$-10\log_{10}\left(\right|S_{21}^{ll}{|}^{2}+|S_{11}^{ll}{|}^{2})$	0.02dB	0.13dB
Metal/dielectrics	$-[10\log_{10}\left(\right|S_{21}{|}^{2}+|S_{11}{|}^{2})-10\log_{10}\left(\right|S_{21}^{ll}{|}^{2}+|S_{11}^{ll}{|}^{2})]$	0.24dB	0.48dB
Total insertion loss	$-10\log_{10}{\left|S_{21}\right|}^{2}$	0.30dB	0.68dB

(*) $S_{11}$ and $S_{21}$ are the measured reflection and transmission coefficients of the CPW line. $S_{11}^{ll}$ and $S_{21}^{ll}$ are the simulated reflection and transmission coefficients of a CPW line model based on lossless media.

## Data Availability

Not applicable.
